# Metabolic state-driven monitoring and control of abnormal pigment formation in sodium gluconate fermentation by *Aspergillus niger*

**DOI:** 10.1016/j.synbio.2026.06.002

**Published:** 2026-07-02

**Authors:** Jingchun Sun, Yuanyuan Jiang, Xing Jiang, Zhen Chen, Xiang Ke, Xiwei Tian, Ju Chu, Feng Xu

**Affiliations:** a State Key Laboratory of Bioreactor Engineering, Qingdao Innovation Institute of East China University of Science and Technology, East China University of Science and Technology, Shanghai, 200237, China; b National Center of Bio-Engineering & Technology (Shanghai), East China University of Science and Technology, Shanghai, 200237, China; c Shanghai Collaborative Innovation Center for Biomanufacturing Technology, East China University of Science and Technology, Shanghai, 200237, China

**Keywords:** Sodium gluconate fermentation, Industrial scale-down modeling, Inorganic salt regulation, Yellow pigment formation, Raman spectroscopy monitoring, Soft sensor

## Abstract

Abnormal pigment formation during late-stage fungal fermentation poses a significant challenge to industrial product quality. This study established an integrated framework to understand and control pigmentation in *Aspergillus niger* sodium gluconate fermentation. First, a metabolism-oriented scale-down strategy, simulating industrial glucose consumption trajectories, confirmed that metabolic rate deterioration drives pigment accumulation. Crucially, a rate-driven soft sensing strategy was developed by integrating online Raman spectroscopy, which provided real-time kinetic inputs to significantly enhance pigment prediction accuracy. Guided by this monitoring framework, inorganic salt composition was optimized to stabilize metabolic activity, effectively suppressing pigment formation. Crucially, fermentation validation confirmed that the strategy effectively suppressed abnormal pigmentation, even under existing imperfect oxygen supply conditions. This work presented a transferable engineering paradigm that combines metabolic characterization, intelligent spectral monitoring, and rational nutritional intervention, offering a robust solution for quality control in large-scale fungal fermentations.

## Graphical abstract

**Figure:**
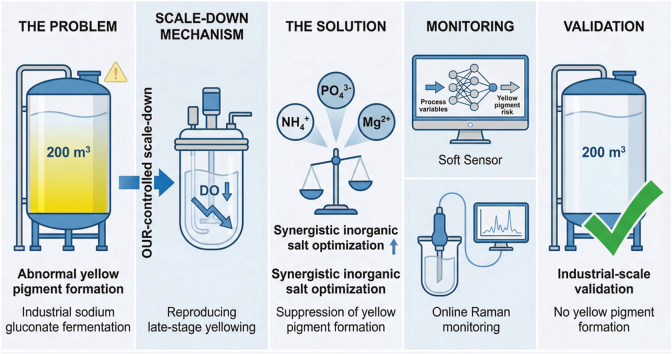

## 1 Introduction

Filamentous fungi are extensively used in industrial fermentation for the production of organic acids, enzymes, and other bulk biochemicals due to their high productivity and scalability [1,2]. Nevertheless, under industrial-scale operation, fungal fermentations frequently exhibit complex late-stage behaviors that are difficult to capture and control at the laboratory scale [3]. Among these behaviors, the unexpected formation of colored byproducts during the later phase of fermentation is commonly observed in various fungal systems and is generally associated with declining cellular activity and metabolic instability [4]. Such pigmentation phenomena are not restricted to a specific organism or product, but rather reflect a broader challenge in managing metabolic state transitions during prolonged cultivation. In industrial practice, these late-stage anomalies are often detected only after they have already affected broth appearance or downstream processing, highlighting the lack of effective predictive indicators [5]. Therefore, establishing a systematic engineering framework to reproduce, monitor, and ultimately mitigate late-stage metabolic abnormalities is of both scientific and practical significance.

A prerequisite for mechanistic understanding and model development is the reliable reproduction of industrial-scale phenomena under controlled laboratory conditions [6]. However, conventional scale-down approaches based on geometric or operational similarity often fail to capture late-stage metabolic behaviors, as these behaviors are primarily governed by cellular metabolic rates rather than absolute process parameters [7,8]. Consequently, there is a need for scale-down strategies that can faithfully reproduce industrial metabolic states, enabling subsequent modeling and control studies. Once industrial phenomena are successfully reproduced at the laboratory scale, data-driven modeling approaches offer a powerful means to quantify and predict process behavior [9]. Soft sensing techniques, which infer difficult-to-measure variables from routinely available process data, have been widely applied in fermentation monitoring [10,11]. However, for complex late-stage phenomena such as pigment formation, conventional soft sensors frequently suffer from limited robustness due to insufficient representation of the underlying metabolic dynamics. Enhancing the physiological relevance of model inputs is therefore essential for improving predictive performance. In this context, spectroscopic techniques, particularly Raman spectroscopy, have attracted increasing attention as non-invasive tools for real-time monitoring of key fermentation variables [12,13]. Raman spectroscopy enables online quantification of substrates, biomass, and products, providing rich process information that can be further transformed into rate-based descriptors reflective of cellular metabolic activity [14]. Integrating Raman-derived information into soft sensing models represents a promising strategy for bridging the gap between observable process variables and latent metabolic states.

Ultimately, predictive modeling alone is insufficient unless it can be translated into effective and economically feasible process interventions [15]. While strategies such as increasing aeration capacity or modifying fermenter configuration can mitigate metabolic stress to some extent, these approaches are often capital-intensive, difficult to retrofit, and lack flexibility during routine industrial operation [16,17]. In contrast, medium composition, particularly inorganic salt formulation, represents a practical and scalable lever for regulating cellular metabolism at the biochemical level without requiring major equipment modification [18]. Inorganic ions play critical roles in enzyme activation, energy metabolism, osmotic balance, and intracellular signal transduction [19]. Subtle variations in the type and concentration of inorganic salts can significantly alter cellular growth kinetics, substrate utilization rates, and overall fermentation performance, thereby influencing the onset and progression of late-stage physiological instability [20]. Importantly, inorganic salt regulation allows targeted modulation of metabolic rates rather than merely compensating for process limitations, offering a more fundamental and controllable approach to mitigating pigment formation.

In this study, we present an integrated engineering strategy for addressing late-stage pigment formation in fungal fermentation, using *Aspergillus niger* (*A. niger*) sodium gluconate production as a representative industrial case. Industrial-scale pigmentation was first reproduced in a laboratory fermenter through a metabolism-oriented scale-down approach. A soft sensing model was then developed to characterize pigment formation dynamics, followed by the incorporation of Raman spectroscopy to enhance model performance via online metabolic information. Guided by insights from the modeling framework, inorganic salt composition was subsequently optimized to regulate metabolic state and suppress pigment formation, and the proposed strategy was finally validated at the industrial scale. This work demonstrates a systematic pathway from phenomenon reproduction to model-based intervention, offering a generalizable framework for managing late-stage metabolic abnormalities in industrial fermentations.

## 2 Methods and materials

### 2.1 Strain and culture condition

The industrial *A*. *niger* FYSC121 strain provided by Shan Dong Fuyang Biological Technology Co., Ltd. was used throughout this study [21]. The spores from stock cultures were transferred using a sterile bamboo scraper and spread evenly onto fresh maintenance slants. These flasks were incubated at 37 °C for 60 h. For seed inoculation, 50 mL of sterile distilled water was added to the mature slant, and spores were gently scraped into suspension. The entire spore suspension was inoculated into the seed bioreactor. The temperature was maintained at 38 °C with an aeration rate of 3.0 vvm. The initial agitation speed was set to 500 rpm and subsequently increased to 800 rpm during the production phase to meet oxygen demand. The pH was controlled at approximately 5.50 using 10 M NaOH. For the 50 L scale-down system, inoculation was performed via differential pressure transfer, moving seed culture from a 30 L seed tank through steam-sterilized transfer piping. The fermentation process was controlled at 38 °C with a constant aeration rate of 1.0 vvm. A distinct agitation strategy was employed: the initial speed was set at 300 rpm, increased to 500 rpm for the first 6 h (lag phase), and then ramped up to 800 rpm from 6 h onwards until the end of fermentation to ensure sufficient oxygen mass transfer and mixing.

Three distinct media formulations were used for strain maintenance, seed preparation, and fermentation: The solid medium contained (per liter): glucose 60 g, urea 0.2 g, KH_2_PO_4_ 0.13 g, MgSO_4_⋅7H_2_O 0.02 g, corn steep liquor 1.0 g, and agar 20 g. After adjusting the pH to 6.5–7.0, 5.0 g/L CaCO_3_ was added. Seed medium: Used for both 5 L and 50 L bioreactors, consisting of (per liter): MgSO_4_⋅7H_2_O 0.19 g, KH_2_PO_4_ 0.5 g, (NH_4_)_2_HPO_4_ 0.19 g, corn steep liquor 2.1 g, and antifoam 0.2 mL. The pH was adjusted to 6.5-7.0 prior to sterilization. Fermentation medium (per liter): glucose 300 g, MgSO_4_⋅7H_2_O 0.2 g, KH_2_PO_4_ 0.17 g, (NH_4_)_2_HPO_4_ 0.25 g, urea 0.2 g, and antifoam 0.2 mL, with pH adjusted to 6.5–7.0.

### 2.2 Analytical methods

Biomass accumulation was assessed by determining the dry cell weight (DCW) [22]. A 10 mL aliquot of the fermentation broth was filtered through pre-weighed qualitative filter paper, washed thoroughly with distilled water to eliminate residual medium components, and dried at 105 °C until a constant weight was achieved. For glucose analysis, the fermentation supernatant was diluted with distilled water to ensure concentrations fell within the linear detection range, and subsequently quantified using an SBA-40E Biosensor Analyzer (Institute of Biology, Shandong Academy of Sciences, China) [22]. Sodium gluconate concentration was determined via High-Performance Liquid Chromatography (HPLC) equipped with a C18 reverse-phase column (4.6 mm × 250 mm,5 μm) and an Ultraviolet (UV) detector [23]. The chromatographic separation was performed using a mobile phase consisting of 5% methanol and 95% aqueous phosphoric acid solution (1.2%), operated at a flow rate of 1.0 mL/min. The detection wavelength was set to 210 nm, with the column temperature maintained at 26°C and an injection volume of 20 μL. All samples were filtered through a 0.22 μm membrane prior to injection. The formation of yellow pigment was quantified spectrophotometrically. Cell-free supernatant was obtained by centrifugation at 8000 rpm for 10 min. The optical density (OD) or transmittance was measured at 405 nm (OD_405_) using a UV-Vis spectrophotometer (Shimadzu UV-2600). Lower transmittance or higher absorbance values indicated higher pigment concentration. Based on the standard quality control (QC) criteria of the cooperating industrial facility, an OD_405_ ≤ 0.30 (corresponding to a broth transmittance ≥50%) is established as the acceptance threshold. Batches exceeding this limit require costly secondary decolorization treatments using activated carbon, while those within the limit can proceed directly to standard downstream crystallization.

The composition of the exhaust gas was monitored online using an Extrel mass spectrometer. The Oxygen Uptake Rate (OUR), Carbon Dioxide Evolution Rate (CER), and Respiratory Quotient (RQ) were calculated using Equations. (1)–(3) [24]:

(1) $$\text{OUR}=\frac{F_{\text{in}}}{V}\left[C_{O_{2}\text{in}}-\frac{C_{\text{inert}\;\text{in}}\cdot C_{O_{2}\text{out}}}{1-\left(C_{O_{2}\text{out}}+C_{{\text{CO}}_{2}\text{out}}\right)}\right]\cdot \frac{273}{273+t_{\text{in}}}\cdot P_{\text{in}}\cdot \frac{1}{1+h}\cdot 10^{-5}$$

(2) $$\text{CER}=\frac{F_{\text{in}}}{V}\left[\frac{C_{\text{inert}\;\text{in}}\cdot C_{{\text{CO}}_{2}\text{out}}}{1-\left(C_{O_{2}\text{out}}+C_{{\text{CO}}_{2}\text{out}}\right)}-C_{{\text{CO}}_{2}\text{in}}\right]\cdot \frac{273}{273+t_{\text{in}}}\cdot P_{\text{in}}\cdot \frac{1}{1+h}\cdot 10^{-5}$$

(3) RQ=CEROUR

Where *F*_in_ represents the inlet aeration rate; *V* is the working volume; $C_{\text{inert}\;\text{in}}$, $C_{O_{2}\text{in}}$, and $C_{{\text{CO}}_{2}\text{in}}$ denote the inlet fractions of nitrogen, oxygen, and carbon dioxide, respectively; $C_{O_{2}\text{out}}$ and $C_{{\text{CO}}_{2}\text{out}}$ are the outlet fractions of oxygen and carbon dioxide; and *P*_in_, *t*_in_, and *h* represent the inlet pressure, temperature, and humidity, respectively.

### 2.3 Data-driven modeling and soft sensor development

#### 2.3.1 Data collection and preprocessing

A dataset comprising 82 sample points was compiled from 8 distinct fermentation batches (including historical industrial data and lab-scale experiments). The raw input variables included fermentation time (t), glucose concentration (S), sodium gluconate concentration (P), and biomass (X). The target variable was the pigment intensity (OD_405_). Data were normalized using Min-Max scaling to a range of [0, 1] to ensure numerical stability during model training. To capture metabolic dynamics, the raw feature space was augmented with derived physiological descriptors: specific growth rate (μ), specific glucose consumption rate (qS), specific production rate (qP), yield coefficient (Y_P/S_), and glucose utilization efficiency (P/S). Correlation analysis (Pearson coefficient) was performed to evaluate the relationships between features and the target variable [25].

#### 2.3.2 Model training and evaluation

Nearly 20 machine learning (ML) regression algorithms were benchmarked using the Python scikit-learn library, including Linear Regression, Support Vector Regression (SVR), Random Forest, XGBoost, and Extra Trees. The dataset was randomly split into training (80%) and testing (20%) sets. A 5-fold cross-validation was applied during hyperparameter tuning (via Grid Search) to prevent overfitting. Model performance was evaluated using the Coefficient of Determination (R^2^) and Mean Squared Error (MSE) [26]. Learning curves were plotted to assess bias-variance trade-offs.

#### 2.3.3 Feature importance analysis

To elucidate the biological drivers underpinning pigment formation and decode the model's decision-making process, we employed SHAP (SHapley Additive exPlanations), a game-theoretic approach that quantifies the marginal contribution of each feature to the model output [27]. The TreeExplainer algorithm (from the shap Python library) was used to compute SHAP values for the best-performing ensemble model.

### 2.4 Raman spectroscopy and chemometric modeling

Raman spectra were acquired online using a RAMINA process analyzer (Thermo Fisher Scientific) equipped with a 785 nm excitation laser. To effectively capture rapid metabolic dynamics and physiological changes in glucose and sodium gluconate levels, spectral data were collected at a high frequency of 15-min intervals. Raw spectral data were preprocessed using the Standard Normal Variate (SNV) transformation. This step was critical to eliminate non-chemical physical variations (such as scattering effects and path length differences) and to enhance chemically relevant spectral features. Principal Component Analysis (PCA) was subsequently performed to reduce data dimensionality and visualize major variance trends [28]. The validity and applicability of the PCA model were assessed using Hotelling's T^2^ statistic and Q-residuals [29]. In addition, leverage and standardized Y-residuals were calculated to diagnose the regression model, allowing for the identification and analysis of high-leverage points and potential outlier samples. Quantitative calibration models linking spectral data to fermentation process variables (specifically sodium gluconate concentration) were constructed using Partial Least Squares Regression (PLSR). PLSR was selected for its proficiency in handling multicollinearity within the spectral dataset by extracting latent variables that maximize covariance with the response variable. The dataset (70 sample points) was randomly partitioned into two subsets: 70% for the training set (calibration) and 30% for the independent testing set (prediction). To rigorously prevent data leakage and avoid overfitting to batch-specific noise, the data split was performed on a batch-wise basis rather than random sampling. The testing set consisted exclusively of data from a completely separate, independent fermentation batch that was not included in the training phase. The training set was used to learn data features and optimize model parameters, while the testing set served to valid the model's generalization capability on unseen data. The performance of the regression models was evaluated based on the Coefficient of Determination (R^2^) and the Root Mean Square Error (RMSE).

### 2.5 Inorganic salt optimization experiment

To investigate the metabolic regulation of pigment formation, single-factor experiments were conducted. Specifically, four inorganic nitrogen sources, ammonium sulfate ((NH_4_)_2_SO_4_), diammonium phosphate ((NH_4_)_2_HPO_4_), ammonium nitrate (NH_4_NO_3_), and ammonium chloride (NH_4_Cl), were tested at equivalent nitrogen molar concentrations. The KH_2_PO_4_ concentrations were varied from 0.59 to 2.36 g/L. The MgSO_4_⋅7H_2_O concentrations were tested at 0.25, 0.5, and 0.7 g/L. Based on single-factor results, a combinatorial optimization experiment was performed in the 50 L scale-down system using the following optimized inorganic salt formulation: 0.6 g/L (NH_4_)_2_HPO_4_, 0.5 g/L KH_2_PO_4_, and 0.333 g/L MgSO_4_⋅7H_2_O. Control fermentations used the unoptimized industrial baseline medium.

### 2.6 Statistical analysis

All fermentation experiments were performed in triplicate. Data are presented as mean ± standard deviation (SD). Statistical significance was determined using one-way Analysis of Variance (ANOVA) followed by Tukey's post-hoc test (p < 0.05) using OriginPro 2024 software.

## 3 Results and discussion

### 3.1 Recapitulation of the industrial-scale yellow pigmentation phenotype in a 50 L scale-down bioreactor

**Fig. 1:**
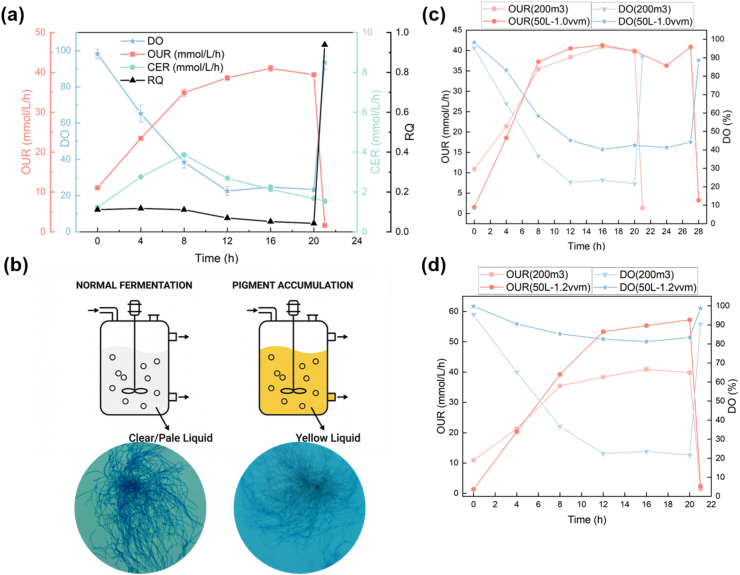
Physiological characterization and multi-scale analysis of the Sodium gluconate fermentation process. (a) Time-course evolution of key respiratory parameters in a 200 m^3^ bioreactor, including Dissolved Oxygen (DO), Oxygen Uptake Rate (OUR), Carbon Dioxide Evolution Rate (CER), and Respiratory Quotient (RQ); (b) Schematic representation and microscopic morphological comparison between normal fermentation and the abnormal pigment accumulation phenotype; (c) Comparative profiles of OUR and DO between the industrial scale (200 m^3^) and the pilot scale (50 L) operating at an aeration rate of 1.0 vvm; (d) Comparative profiles of OUR and DO between the industrial scale (200 m^3^) and the pilot scale (50 L) at an increased aeration rate of 1.2 vvm.

Fig. 1a illustrates the typical profile of industrial-scale sodium gluconate fermentation in a 200 m^3^ bioreactor. Under standard operating conditions, the process concluded within approximately 20 h, achieving a maximum glucose consumption rate of 20 g/L/h. Throughout the majority of the fermentation, the conversion yield of glucose to sodium gluconate remained high and stable (0.90–0.94 mol/mol), indicating an efficient and well-regulated primary metabolism. However, despite this favorable kinetic performance, abnormal yellow pigmentation of the broth frequently occurred during the late stage of industrial production (Fig. S1). Statistical analysis of multiple industrial batches revealed that this pigmentation was exclusively associated with the terminal phase of fermentation, rather than the active growth or acid-production phases. This accumulation of yellow pigment significantly compromised the visual quality of the product and imposed a heavy burden on downstream purification, thereby increasing production costs and reducing process robustness.

Microscopic examination provided further insights into the physiological state of *A*. *niger* by comparing batches with and without pigment formation (Fig. 1b). In non-pigmented batches, the mycelia exhibited dense, robust, and filamentous structures with strong staining affinity, reflecting high physiological integrity. In contrast, biomass from pigmented batches was characterized by dispersed mycelial fragments and poor staining properties. The morphological deterioration suggested a strong correlation between pigment formation and cell senescence, likely induced by adverse physiological conditions in the late fermentation stage. In addition, a more detailed analysis of the tail gas mass spectrometry data provides deeper mechanistic insights into this late-stage deterioration. The late fermentation stage was characterized by a sharp decline in the OUR, while the CER remained disproportionately elevated, leading to an abnormal shift in the RQ (Fig. 1a). The distinct physiological signature indicated a severe decoupling of oxidative metabolism. Under these oxygen-limited conditions, although the specific substrate uptake rate (qS) may remain active, the efficient oxidation of the carbon source was compromised. The metabolic decoupling drastically reduced energy (ATP) generation efficiency, rendering the cells unable to meet their increasing maintenance energy demands. Overall, the results demonstrated that the energy shortage might trigger premature cellular senescence and the subsequent activation of stress-induced secondary metabolic pathways, culminating in yellow pigment formation.

To verify the hypothesis and facilitate systematic mechanistic studies, we employed a rational scale-down strategy to reproduce the industrial-scale yellowing phenomenon in a laboratory-scale 50 L bioreactor (Fig. 1c). The scale-down criterion was based on prior physiological studies of *A. niger*, which established the on-line OUR as a reliable indicator of glucose consumption and sodium gluconate production rates. Therefore, the scale-down protocol was designed to match the glucose consumption rate of the acid-production phase with the average OUR levels observed during the stable phase of industrial fermentation. When the agitation speed and aeration rate in the 50 L bioreactor were set to 450 rpm and 1.0 vvm., respectively, the laboratory-scale fermentation achieved a maximum glucose consumption rate of 20 g/L/h, closely mirroring the kinetics of the 200 m^3^ industrial vessel (Fig. 1c). Crucially, under these conditions, yellow pigment formation was successfully reproduced in the late stage, confirming that the scale-down model captured not only the kinetic characteristics but also the undesirable industrial phenotype. To further elucidate the role of oxygen supply, the aeration rate in the 50 L bioreactor was increased to 1.2 vvm (Fig. 1d). while maintaining constant agitation, simulating an increase in air compressor power at the industrial scale. The adjustment shortened the fermentation time from 28 h to 24 h and effectively mitigated yellow pigment formation. These results indicated that enhanced oxygen supply sustained primary metabolic activity, promoted cell maintenance, and delayed the onset of senescence-associated pigmentation. In addition, these findings validated the reliability of the OUR-based scale-down method and highlighted the pivotal role of oxygen availability in regulating the late-stage physiological state of *A. niger*. The validated model could serve as a robust experimental platform for subsequent metabolic regulation and process optimization studies.

Although increasing aeration effectively suppressed yellow pigment formation in the scale-down system, this strategy alone does not provide a universal or proactive solution for industrial operation. Oxygen supply represents an external control variable that primarily compensates for metabolic deterioration after it has already emerged, rather than identifying the onset of physiological instability at an early stage. Moreover, excessive aeration is often constrained by energy consumption, equipment capacity, and shear sensitivity, limiting its applicability as a routine control strategy in large-scale fermentation [30,31]. More importantly, the aeration experiments revealed that yellow pigment formation is tightly coupled to late-stage metabolic transitions rather than to a single operational parameter. This observation underscores the necessity of developing predictive tools capable of quantitatively characterizing the physiological state of the culture before irreversible pigment accumulation occurs. Such tools would enable early diagnosis of metabolic instability and provide actionable guidance for subsequent process intervention, rather than relying on reactive operational adjustments. Therefore, following the successful reproduction of industrial pigmentation behavior and its mitigation via oxygen enhancement, we next focused on establishing a data-driven soft sensing framework for real-time pigment prediction.

### 3.2 Development of a data-driven soft sensor for pigment prediction

#### 3.2.1 Soft sensor variable selection based on physiological correlation

The rigorous selection of input variables is a prerequisite for developing robust and interpretable soft sensing models. Yellow pigment accumulation in *A. niger* is intrinsically linked to the late-stage physiological status of the culture, governed specifically by metabolic activity, carbon flux dynamics, and biomass maintenance. In addition, the fermentation time, biomass, glucose, and sodium gluconate concentrations were designated as the core predictors. Fermentation time acts as a temporal proxy for physiological senescence, a factor explicitly correlated with pigment onset in our scale-down validation. Biomass concentration functions as a metric of cellular growth and viability, whereas glucose concentration quantifies substrate availability relative to metabolic demand. Furthermore, sodium gluconate concentration captures the cumulative output of primary metabolism, serving as an indirect gauge of the metabolic burden imposed on the cellular machinery. We benchmarked 19 ML algorithms, ranging from linear regression to complex ensemble methods and kernel-based approaches (Fig. S2a). The results revealed a severe lack of generalization capability across almost all algorithms. While some models (e.g., CatBoost, XGBoost) achieved reasonable scores on the training set (blue bars), their performance on the testing set (pink bars) was dismal, with R^2^ values frequently dropping near zero or becoming negative (e.g., Gaussian Process). The best-performing model in this initial screening was the Support Vector Regressor with an RBF kernel (SVR RBF), achieving an RMSE of 0.093 (Fig. S2b). However, the majority of models hovered around a mean RMSE of 0.134, indicating significant prediction errors. The catastrophic failure of the Gaussian Process model (RMSE >0.5) and the generally poor testing scores suggested that raw trajectory data alone lacked sufficient information density to capture the complex, non-linear dynamics of secondary metabolite formation. The models were essentially memorizing the training data rather than learning the underlying metabolic rules, leading to severe overfitting.

#### 3.2.2 Enhancing model robustness via biological feature engineering

**Fig. 2:**
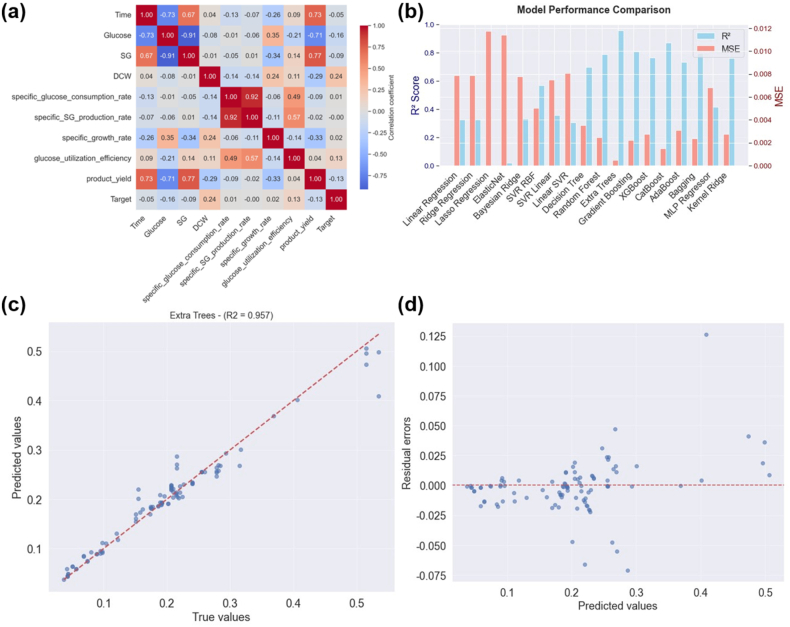
Feature correlation analysis and comprehensive evaluation of machine learning models for soft sensor development. (a) Pearson correlation matrix heatmap illustrating the linear interdependencies among fermentation process variables and the target variable. The color scale ranges from dark blue (strong negative correlation) to dark red (strong positive correlation). (b) Comparative performance benchmarking of various regression algorithms. The primary axis (blue bars) represents the Coefficient of Determination (R^2^), while the secondary axis (red bars) represents the Mean Squared Error (MSE). (c) Parity plot for the optimized Extra Trees model on the testing set (R^2^ = 0.957). The red dashed diagonal line represents the ideal prediction (y = x), indicating a high degree of agreement between model outputs and actual measurements. (d) Residual analysis plot showing the distribution of prediction errors against predicted values. The random scatter of data points around the horizontal zero line (red dashed) confirms the absence of significant systematic bias in the model.

Recognizing that raw variables fail to reflect physiological states, a domain-knowledge-driven feature engineering strategy was implemented [32]. The feature space was expanded by deriving secondary kinetic parameters that explicitly described the metabolic activity of *A. niger*. These engineered features included: Specific growth rate (μ), Specific glucose consumption rate (qS), Specific product formation rate (qP), Glucose utilization efficiency (P/S), and Product yield (Y_P/S_). This transformation aimed to convert static concentration data into dynamic rate information, providing the algorithms with deeper insights into the velocity and efficiency of the cellular machinery. To elucidate the complex interdependencies among fermentation parameters and identify key predictors for the soft sensor model, a Pearson correlation analysis was conducted (Fig. 2a). The analysis revealed distinct metabolic signatures, most notably the strong negative correlation between residual glucose and sodium gluconate (r = −0.91), which reflects the stoichiometric conversion of substrate to product. Furthermore, a high degree of collinearity was observed between the specific glucose consumption rate and the specific product formation rate (r = 0.92), indicating a tight kinetic coupling between catabolic and anabolic activities. Crucially, the target variable (pigment intensity) exhibited only weak linear correlations with macroscopic state variables; the highest positive correlation was observed with biomass (r = 0.24), while associations with glucose (−0.16) and product concentration (−0.09) were negligible. This lack of significant linear dependence underscores the complexity of the pigmentation mechanism and provides a compelling justification for employing non-linear machine learning algorithms, rather than simple linear regression, to accurately capture the physiological shifts associated with the yellowing phenotype. Following feature augmentation, the model selection process was repeated (Fig. 2b). The impact of feature engineering was transformative. Unlike the initial screening where models struggled to generalize, the tree-based ensemble methods, specifically Extra Trees model demonstrated a dramatic improvement in predictive accuracy. The optimized Extra Trees model achieved a testing dataset R^2^ of 0.957, a significant leap from the baseline performance of raw-variable models (Fig. 2c). As detailed in the residual analysis, the model predictions clustered tightly around the observed values with unbiased error distribution (Fig. 2d).

The superiority of the Extra Trees model after feature engineering can be attributed to its ability to utilize the kinetic signals embedded in the derived features. Feature importance analysis (Fig. S3a) confirmed that while Time and Glucose remained fundamental, the model heavily relied on DCW and Specific glucose consumption rate to fine-tune its predictions. This directly aligns with the physiological mechanisms identified from the tail gas analysis (Section 3.1). The model's heavy reliance on the qS and DCW confirms that pigment formation is not governed by static concentrations, but rather by the dynamic imbalance between substrate uptake and oxidative capacity. When qS remains high but oxidative metabolism is decoupled (as evidenced by declining OUR), the resulting failure to satisfy maintenance energy demands triggers the stress response leading to pigmentation. By capturing this kinetic uncoupling, the soft sensor achieves high mechanistic interpretability alongside predictive accuracy. Furthermore, learning curve analysis (Fig. S3b) demonstrated rapid convergence of training and validation errors, contrasting sharply with the overfitting observed in the raw-data models (Fig. S2a). This confirmed that the engineered features successfully bridged the gap between observable process data and the hidden physiological state of pigmentation, resulting in a robust, high-precision soft sensor suitable for industrial application.

In addition, the results indicated that yellow pigment formation was fundamentally governed by cellular metabolic kinetics rather than static state variables. By transforming conventional concentration-based measurements into rate-oriented descriptors, the soft sensing model was able to capture the latent physiological state associated with metabolic stress and pigmentation. However, despite the improved robustness achieved through feature engineering, the current framework still relied on offline or intermittently measured inputs, which inherently limited its responsiveness and practical applicability for real-time process monitoring and intervention [33]. From an engineering perspective, the effectiveness of the soft sensor is therefore constrained not by model structure, but by the availability and timeliness of physiologically meaningful input variables. Specifically, key kinetic indicators such as biomass growth rate and substrate consumption rate cannot be continuously obtained using traditional analytical methods, creating a gap between the modeled metabolic state and real-time process dynamics. This limitation motivates the integration of online analytical techniques capable of providing continuous, high-resolution measurements that can be directly translated into metabolic rate information.

### 3.3 Integration of online Raman spectroscopy for intelligent process monitoring

#### 3.3.1 Raman spectral fingerprinting of fermentation dynamics

**Fig. 3:**
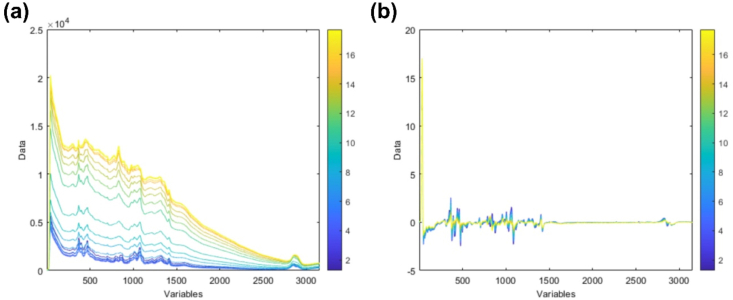
Comparison of raw and preprocessed spectral data used for soft sensor modeling. (a) Original spectral profiles collected during the fermentation process. The x-axis represents the wavenumbers (cm^−1^), and the color scale (from blue to yellow) indicates the progression of sample index. (b) Preprocessed spectral data after applying Standard Normal Variate (SNV) transformation.

Raman spectroscopy offers a unique opportunity to bridge this gap by enabling non-invasive, real-time quantification of substrates, products, and biomass in complex fermentation systems. When combined with appropriate chemometric modeling, Raman-derived concentrations can be further converted into kinetic features that align directly with the rate-based soft sensing framework developed in this study. Consequently, integrating Raman spectroscopy as an upstream data source transforms the soft sensor from an offline diagnostic tool into an intelligent, metabolism-aware monitoring system, capable of tracking physiological state transitions associated with pigment formation in real time. Despite the complex matrix of the fermentation broth, containing biomass, residual substrates, and suspended solids, the raw spectra exhibited distinct signature peaks attributable to sodium gluconate (Fig. 3). However, quantitative analysis was complicated by two factors [1]: the structural similarity between glucose and sodium gluconate, leading to significant spectral overlap; and [2] physical interferences such as bubble scattering and biomass heterogeneity. To mitigate these effects, raw spectra were preprocessed using the Standard Normal Variate (SNV) transformation [34]. This normalization effectively minimized baseline shifts and multiplicative scattering effects, thereby enhancing chemically relevant information. The SNV-treated spectra displayed improved consistency in peak morphology across fermentation time points, particularly in the fingerprint region (150–1500 cm^−1^), facilitating the deconvolution of substrate, product, and biomass signals (Fig. 5b).

Subsequently, principal component analysis (PCA) was employed to reduce dimensionality and visualize the underlying metabolic trajectories (Fig. S4). The first principal component (PC1) captured the majority of variance for biomass (53.59%), glucose (55.61%), and sodium gluconate (55.64%), indicating a highly coordinated metabolic evolution. The cumulative explained variance for the first two PCs exceeded 70% for all variables (Sodium gluconate: 75.30%; Glucose: 70.15%; Biomass: 63.66%), confirming the robustness of the low-dimensional representation. Prior to quantitative PLS modeling, rigorous outlier detection and data cleaning procedures were performed to ensure the integrity of the spectral dataset. The multivariate diagnostic results confirmed the absence of influential outliers and the high suitability of the dataset for subsequent regression modeling (detailed chemometric diagnostic plots are provided in Supplementary Fig. S5).

#### 3.3.2 Quantitative modeling and validation

**Fig. 4:**
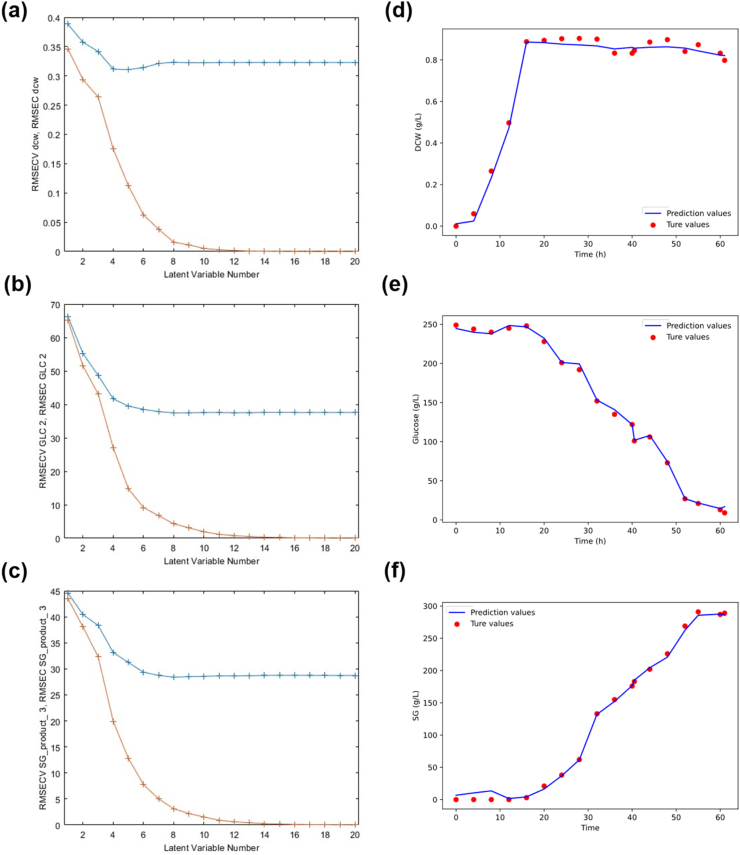
Optimization of latent variable numbers and validation of prediction performance for key fermentation parameters. (a–c) Determination of optimal Latent Variable (LV) numbers: The plots display the trends of Root Mean Squared Error of Calibration (RMSEC, orange line) and Root Mean Squared Error of Cross-Validation (RMSECV, blue line) as a function of the number of latent variables. The optimal number of LVs was selected based on the minimum RMSECV to avoid overfitting. (a) Model for Dry Cell Weight (DCW). (b) Model for Glucose concentration (GLC). (c) Model for Sodium Gluconate (SG). (d–f) Time-course prediction profiles on the testing dataset: Comparison between the model predicted values (blue solid lines) and the experimental true values (red dots) throughout the fermentation process. (d) Prediction trajectory for DCW. (e) Prediction trajectory for Glucose consumption. (f) Prediction trajectory for SG production accumulation. The close alignment between the red dots and blue lines indicates the high generalization capability and robustness of the developed models.

PLS regression models were developed to quantify key fermentation parameters. The optimal number of latent variables (LVs) was determined by analyzing the Root Mean Square Error of Cross-Validation (RMSECV). Although the target components are limited to three, the dynamic fermentation broth introduces severe optical complexities, including variable light scattering due to shifting mycelial morphology, changing viscosity, and background fluorescence from trace metabolites. The prediction error decreased rapidly as the initial LVs captured the primary chemical variance of the targets, and stabilized around 10 components (Fig. 4). The higher-order LVs were mathematically essential to model and suppress the complex, time-varying background matrix. Consequently, 10 LVs were selected to balance model complexity and generalization capability, preventing overfitting. Table 1 summarizes the model metrics: all models achieved high determination coefficients and low RMSE in calibration. Crucially, the sodium gluconate model exhibited superior performance in external validation, underscoring the high specificity of Raman scattering for the target product. To elucidate the chemical basis of the predictions, Variable Importance in Projection (VIP) scores were calculated. For biomass prediction, high VIP scores were clustered in the 1200–1500 cm^−1^ region, likely corresponding to C–H deformation and amide bands associated with cellular proteins and nucleic acids (Fig. S6a) [34]. Conversely, prediction of glucose and sodium gluconate relied heavily on the 500–1000 cm^−1^ region, a range characteristic of C–C and C–O stretching vibrations in carbohydrates (Fig. S6b and S6c) [12]. This spectral specificity confirmed that the PLS models were driving predictions based on genuine biochemical signals rather than spurious correlations. The robustness of the Raman-based monitoring platform was rigorously tested against an independent fermentation batch. Because the testing batch was entirely separate from the training data, the exceptional predictive accuracy achieved, with testing dataset R^2^ values of 0.9929 (Biomass), 0.9983 (Glucose), and 0.9978 (Sodium Gluconate) (Fig. 4d–f). The results demonstrated that the 10-LV PLS models successfully extracted the true chemical variance rather than memorizing batch-specific noise.

**Fig. 5:**
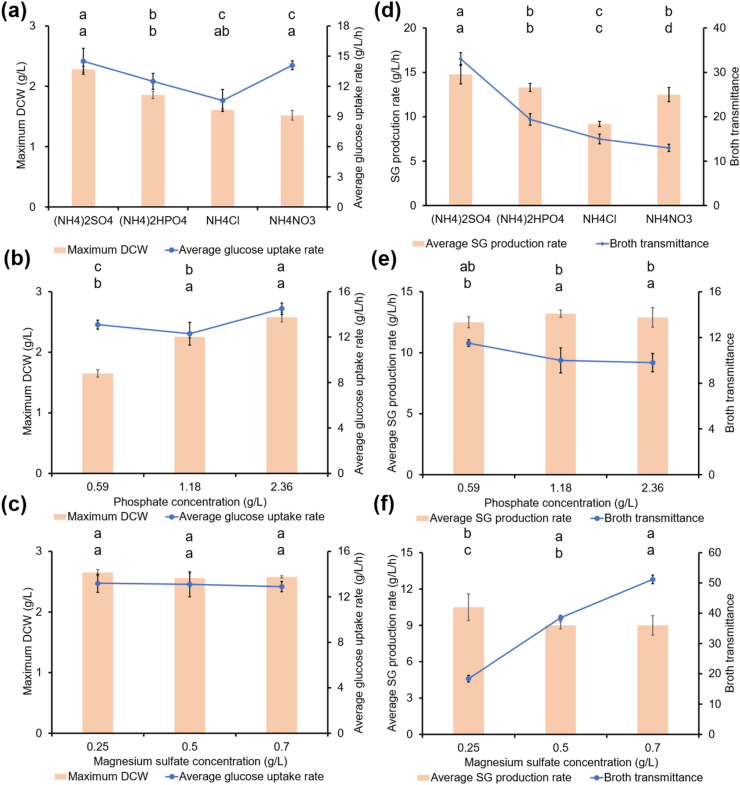
Effects of nitrogen sources, phosphate levels, and magnesium sulfate concentrations on cell growth, glucose metabolism, product synthesis, and broth pigmentation. (a–c) Impact on cell growth and glucose consumption: (a) Influence of different nitrogen sources [(NH_4_)_2_SO_4_, (NH_4_)_2_HPO_4_, NH_4_Cl, NH_4_NO_3_] on maximum dry cell weight (DCW) and average glucose uptake rate. (b) Influence of varying phosphate concentrations (0.59, 1.18, 2.36 g/L) on maximum DCW and glucose uptake rate. (c) Influence of varying magnesium sulfate concentrations (0.25, 0.5, 0.7 g/L) on maximum DCW and glucose uptake rate. (d–f) Impact on product formation and broth quality: (d) Effect of different nitrogen sources on average sodium gluconate (SG) production rate and broth transmittance (an indicator of pigment intensity). (e) Effect of varying phosphate concentrations on SG production rate and broth transmittance. (f) Effect of varying magnesium sulfate concentrations on SG production rate and broth transmittance. Error bars represent the standard deviation of triplicate experiments (n = 3). Different lowercase letters (a, b, c) indicate statistically significant differences among groups (p < 0.05) based on one-way ANOVA followed by Tukey's post-hoc test. Note the significant correlation between magnesium sulfate concentration and broth transmittance in (f), indicating its critical role in pigment formation.

**Table 1:** Evaluation metrics for three key parameters across different datasets.

Matrices	Training dataset R^2^	Training Set RMSE (g/L)	Validation dataset R^2^	Validation dataset RMSECV (g/L)
DCW	0.9936	0.0252	0.9842	0.0512
Glucose	0.9956	14.8127	0.9856	19.4918
Sodium Gluconate	0.9974	12.8094	0.9828	14.2296

Taken together, the Raman-based PLS models established in this study provided a reliable and chemically interpretable means of tracking the core metabolic variables governing the fermentation process. More importantly, the high predictive accuracy achieved for biomass, glucose, and sodium gluconate enabled these traditionally concentration-based measurements to be further transformed into rate-oriented descriptors, such as substrate consumption rate and biomass-specific metabolic activity. These kinetic indicators were directly linked to the physiological state of the culture and were therefore highly relevant to the onset of late-stage metabolic instability. The availability of accurate, online-resolved metabolic information fundamentally changed how pigment formation can be interpreted and addressed. Rather than treating yellow pigmentation as an isolated quality defect, the Raman-enhanced monitoring framework revealed it as a downstream manifestation of altered metabolic flux distribution and reduced cellular maintenance capacity. This insight established a clear quantitative bridge between observable fermentation variables and the hidden physiological processes driving pigment synthesis. With this metabolism-aware monitoring capability in place, the focus can be shifted from passive observation to rational process intervention. Specifically, medium composition, particularly inorganic salt formulation, offered a practical and scalable lever to modulate metabolic rates and flux allocation without altering reactor hardware. Guided by the kinetic information extracted from Raman-enabled models, inorganic nutrient optimization can be designed to stabilize cellular metabolism, delay physiological deterioration, and thereby suppress pigment formation at its source.

### 3.4 The impact of inorganic salts on physiological metabolism and yellow pigment formation

#### 3.4.1 Impact of inorganic nitrogen sources on fermentation kinetics and pigment formation

**Fig. 6:**
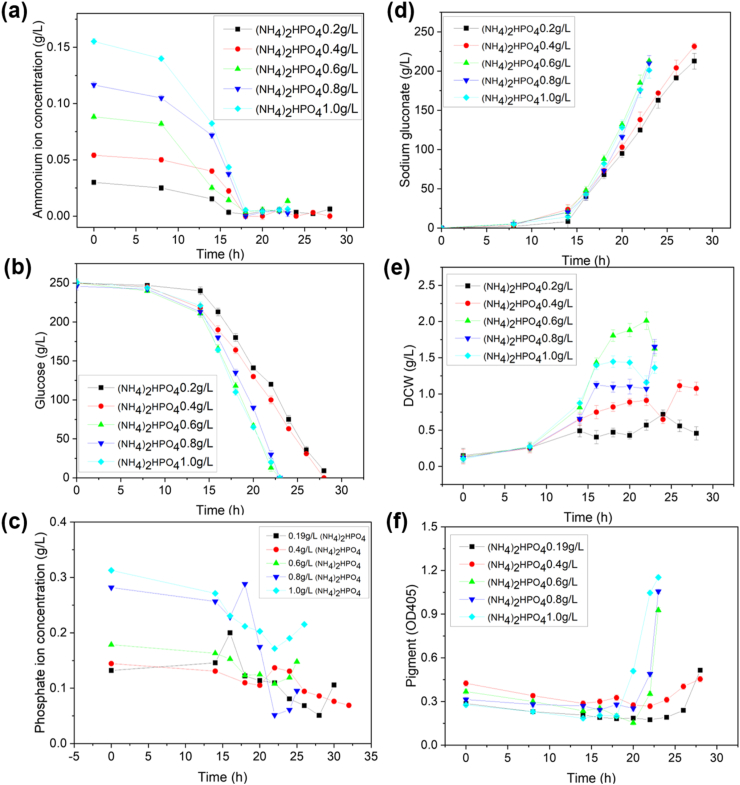
Time-course kinetic profiles of fermentation parameters under varying initial concentrations of (NH_4_)_2_HPO_4_. The fermentation process was monitored at varying supplementation levels ranging from 0.2 g/L to 1.0 g/L (a–c) Nutrient consumption dynamics: (a) Residual ammonium ion concentration. (b) Residual glucose concentration, showing accelerated consumption rates at higher phosphate/nitrogen levels. (c) Residual phosphate ion concentration. (d–e) Growth and production performance: (d) Sodium gluconate titer accumulation. (e) Cell growth curves expressed as Dry Cell Weight (DCW), indicating a positive correlation between nutrient availability and biomass. (f) Product quality indicator: (f) Pigment accumulation intensity measured by optical density at 405 nm (OD_405_). Note that while higher concentrations of (NH_4_)_2_HPO_4_ promote biomass accumulation (e) and glucose consumption (b), they also trigger a sharp increase in pigment formation (f) during the late fermentation phase (after 20 h), particularly at concentrations above 0.8 g/L. Error bars indicate standard deviation (n = 3).

The yellow pigment formation was negatively correlated with fermentation time and with glucose and sodium gluconate concentrations, while exhibiting only a weak dependence on biomass (Fig. 2a). The results indicated that pigment accumulation was primarily linked to metabolic exhaustion rather than biomass proliferation. Accordingly, regulation of cellular metabolic activity via inorganic salt composition represented a rational strategy for stabilizing biomass physiology and mitigating pigment formation. To elucidate how inorganic nutrients modulate the physiological state of *A. niger* and influence pigment synthesis, the effects of various nitrogen sources were investigated firstly. Given the pivotal role of nitrogen metabolism in fungal growth and energy allocation, fermentations were conducted using ammonium sulfate ((NH_4_)_2_SO_4_), diammonium phosphate ((NH_4_)_2_HPO_4_), ammonium nitrate (NH_4_NO_3_), and ammonium chloride (NH_4_Cl) at equivalent nitrogen concentrations (Fig. 6a).

Significant disparities in growth kinetics were observed among the tested sources. Ammonium sulfate supported the most vigorous growth, achieving a maximum biomass of 2.28 g/L at 14–15 h, followed by (NH_4_)_2_HPO_4_ (1.86 g/L) (Fig. 6a). The superior performance was likely attributed to the provision of sulfate, an essential precursor for sulfur-containing amino acids and protein synthesis [35]. Conversely, ammonium nitrate resulted in a markedly lower biomass (1.5 g/L), presumably due to the energetic burden associated with the reduction of nitrate to ammonium prior to assimilation [36]. Ammonium chloride yielded the slowest biomass accumulation, indicating limited suitability under these conditions.

Fermentation kinetics mirrored these growth trends. The ammonium sulfate group exhibited the highest average glucose consumption rate (14.5 g/L/h), outperforming the ammonium chloride group by approximately 37%. Crucially, nitrogen source selection profoundly influenced pigment dynamics. While all groups started with comparable broth clarity, the onset of yellowing varied significantly. Pigmentation commenced earliest in the (NH_4_)_2_HPO_4_ and ammonium nitrate groups (6 h), followed by the ammonium chloride group (12 h). In contrast, ammonium sulfate delayed pigmentation onset to 15 h and maintained the highest broth transmittance (32.5%) at harvest. These findings suggested that ammonium sulfate optimized primary metabolism while simultaneously suppressing the premature activation of secondary metabolic pathways associated with pigmentation. Interestingly, the rapid pigmentation observed in the (NH_4_)_2_HPO_4_ group pointed to a potential regulatory role of phosphate ions, prompting a focused investigation into phosphate concentration.

#### 3.4.2 Regulation of fermentation performance by phosphate concentration

Phosphate serves as a critical component in energy transduction, nucleic acid synthesis, and metabolic regulation [37]. The effect of phosphate ion concentration (0.59–2.36 g/L) on process performance was subsequently evaluated (Fig. 6b & e). A positive correlation was established between phosphate availability and biomass accumulation, with maximum biomass increasing from 1.65 to 2.58 g/L across the tested range. Correspondingly, glucose consumption rates improved from 12.3 to 14.5 g/L/h, confirming that adequate phosphate supplementation enhances metabolic activity. Regarding pigmentation, while total pigment yield remained relatively constant, the kinetics of formation were distinct. Low phosphate conditions triggered rapid yellowing within the first 10 h. Conversely, higher phosphate concentrations delayed the onset of pigmentation to 15 h. This delay is mechanistically consistent with the role of phosphate in central carbon metabolism. High phosphate levels are known to repress the pentose phosphate pathway while enhancing glycolytic flux, thereby channeling carbon towards primary metabolites rather than secondary biosynthetic precursors [38]. Thus, phosphate concentration acted as a temporal regulator, governing the physiological transition from growth to secondary metabolism.

#### 3.4.3 Suppression of pigment formation by magnesium ions

Finally, the influence of magnesium sulfate concentration (0.25, 0.5, and 0.7 g/L) was further examined, given the role of magnesium as a ubiquitous cofactor in enzymatic reactions (Fig. 6c & f). Within the tested range, magnesium concentration exerted a minor influence on cell growth and substrate uptake. Maximum biomass (1.30–1.40 g/L) and glucose consumption rates (12.9–13.2 g/L/h) exhibited slight declines at the highest concentration (0.7 g/L), suggesting a marginal inhibitory effect of excess magnesium on metabolic activity. However, the impact on pigmentation was profound. Increasing magnesium concentration significantly delayed the onset of yellowing. At 0.7 g/L, pigment formation was postponed to 17 h, nearly 4 h later than in low-magnesium conditions. Consequently, the final broth transmittance reached 50%, a 2- to 3-fold improvement over the low-magnesium group. These results indicated that elevated magnesium levels effectively suppressed pigment biosynthesis, likely through the modulation of intracellular ionic homeostasis or specific enzymatic activities involved in secondary metabolism. However, the trade-off between pigment suppression and slight growth inhibition necessitated careful optimization of magnesium dosage.

### 3.5 Synergistic optimization of inorganic salts for pigment elimination

The single-factor analyses revealed that nitrogen, phosphate, and magnesium ions exerted distinct yet interconnected effects on the physiology, fermentation performance, and pigmentation of *A. niger*. A fundamental conflict existed: while high nitrogen and phosphate levels were essential for driving biomass growth and metabolic activity, their excessive stimulation accelerated the onset of senescence-associated pigmentation. Conversely, elevated magnesium levels suppressed pigment formation but at the cost of reduced product biosynthesis rates. These antagonistic effects necessitated a combinatorial optimization strategy to strike a precise balance between maintaining robust primary metabolism and inhibiting secondary metabolite accumulation. To this end, a synergistic inorganic salt strategy was formulated. The optimized concentration of (NH_4_)_2_HPO_4_ was set at 0.6 g/L, which supported rapid biomass accumulation without inducing the metabolic inhibition associated with phosphate excess (Fig. 6). This level provided sufficient catalytic capacity for glucose oxidation while avoiding the metabolic stress that triggers premature secondary metabolism. Additionally, 0.5 g/L KH_2_PO_4_ was included to further facilitate glucose utilization and cell maintenance (Fig. S7). However, results indicated that phosphate stimulation alone was insufficient to completely arrest pigment biosynthesis; in fact, unchecked phosphate-driven flux might accelerate physiological aging if not counterbalanced by other regulatory factors. The critical stabilizing factor in this formulation was the optimized supplementation of MgSO_4_. While the preliminary data indicated that high magnesium (0.7 g/L) effectively suppressed pigmentation, it concurrently penalized sodium gluconate productivity (Fig. S7). Recognizing this trade-off, the MgSO_4_ concentration was recalibrated to 0.333 g/L in the final combinatorial design. As a cofactor for key glycolytic enzymes (e.g., via Mg^2+^-ATP complexes), magnesium ensured stable energy metabolism and reduced the metabolism linked to pigment precursors [39]. Consequently, the final synergistic formulation, comprising 0.6 g/L (NH_4_)_2_HPO_4_, 0.5 g/L KH_2_PO_4_, and 0.333 g/L MgSO_4_, achieved the ideal physiological equilibrium.

**Fig. 7:**
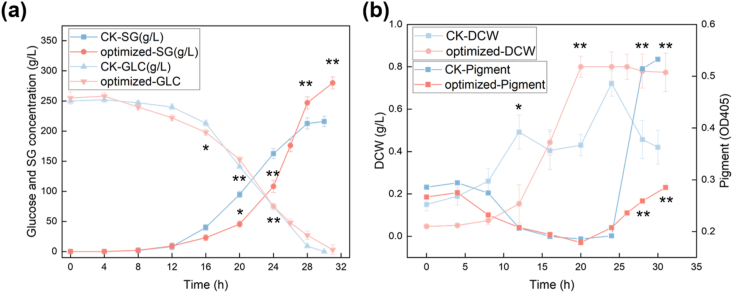
Fermentation performance between the control group (CK) and the optimized strategy in a 50 L bioreactor. (a) Time-course profiles of glucose consumption and sodium gluconate (SG) production. (b) Cell growth and product quality: Time-course profiles of Dry Cell Weight and pigment accumulation. Error bars represent the standard deviation of triplicate experiments (n = 3). Notably, there was no significant statistical difference (p > 0.05) in the final sodium gluconate output between the optimized group and the CK group, indicating that the strategy suppresses pigmentation without adverse effects on primary production.

Finally, to validate the scalability of the integrated strategy (combining optimized inorganic salts and online monitoring), the complete framework was deployed in a 50 L bioreactor designed to simulate the imperfect mixing and oxygen transfer limitations of the 200 m^3^ scale (Fig. 7). The Pigment OD_405_ in the optimized group remained at a low baseline level of approximately 0.2, which was significantly lower than that of the control group. Crucially, the optimized value was safely below the strict industrial quality control threshold of OD_405_ ≤ 0.30. The results confirmed that the targeted nutritional intervention not only effectively suppressed the abnormal accumulation of yellow pigment, but practically ensured that the final broth passed industrial QC standards, thereby eliminating the need for costly and yield-reducing secondary decolorization steps in downstream processing. In stark contrast, the control group far exceeded this critical threshold. Crucially, statistical analysis of the main production indicators confirmed that the optimization strategy did not exert any adverse effects on fermentation efficiency. There was no significant difference (p > 0.05) in the final sodium gluconate titer between the optimized group and the control group (CK), and the overall glucose-to-product yield remained consistently high. The results demonstrated that the synergistic inorganic salt formulation successfully decoupled secondary pigment biosynthesis from primary metabolism, inhibiting the targeted quality defect without penalizing the primary productivity. In addition, while the fermentation was driven by the statically optimized inorganic salt formulation, the soft sensor actively monitored the dynamic physiological state. Its continuous predictions quantitatively verified that the nutritional intervention successfully maintained the cells in a stable trajectory, effectively preventing the kinetic deterioration that typically precedes pigment accumulation.

## 4 Conclusion

In this study, an industrially relevant framework was established to understand and control abnormal pigment formation in *A. niger* sodium gluconate fermentation from the perspective of physiological state. A metabolism-oriented scale-down strategy based on glucose consumption behavior successfully reproduced late-stage yellow pigmentation observed in 200 m^3^ industrial bioreactors, demonstrating that pigment formation originates from metabolic rate deterioration rather than process-specific anomalies. A rate-driven soft sensing model was subsequently developed to link pigment formation with metabolic kinetics. To overcome the limitations of offline measurements, online Raman spectroscopy was integrated to enable real-time quantification of biomass, glucose, and sodium gluconate, thereby providing reliable kinetic inputs, and significantly enhancing the robustness of pigment prediction. Guided by the metabolism-aware monitoring framework, inorganic salt composition was rationally optimized to stabilize cellular metabolism and suppress pigment formation without compromising fermentation performance. The effectiveness of the integrated strategy was validated at the pilot scale under simulated industrial oxygen supply conditions, where abnormal pigmentation was successfully suppressed. Future studies will focus on deploying this integrated strategy directly in 200 m^3^ scale industrial bioreactors to obtain comprehensive industrial operational data. Overall, this work presents a scalable and transferable engineering paradigm that combines metabolic state characterization, intelligent monitoring, and rational intervention, offering a practical solution for improving robustness and quality control in large-scale fungal fermentations. Currently, the Extra Trees-based soft sensor serves as a robust real-time diagnostic tool to validate the physiological efficacy of these static nutritional interventions. Future research will focus on integrating this dynamic risk predictor with automated feeding systems to enable true closed-loop, real-time feedback control of the fermentation trajectory in industrial bioreactors.

## CRediT authorship contribution statement

**Jingchun Sun:** Data curation, Formal analysis, Investigation, Methodology, Validation, Writing – original draft. **Yuanyuan Jiang:** Data curation, Formal analysis, Investigation. **Xing Jiang:** Data curation, Formal analysis. **Zhen Chen:** Data curation, Formal analysis. **Xiang Ke:** Data curation, Formal analysis. **Xiwei Tian:** Conceptualization, Data curation, Funding acquisition, Project administration, Supervision, Writing – review & editing. **Ju Chu:** Data curation, Funding acquisition, Supervision, Writing – review & editing. **Feng Xu:** Conceptualization, Data curation, Project administration, Supervision, Writing – review & editing.

## Declaration of competing interest

The authors declare that they have no known competing financial interests or personal relationships that could have appeared to influence the work reported in this paper.
