# Supplementary material for: Metabolic state-driven monitoring and control of abnormal pigment formation in sodium gluconate fermentation by Aspergillus niger
Source: Synth Syst Biotechnol. 2026 Jul 2;16:1–12. doi: 10.1016/j.synbio.2026.06.002 (PMC13351394; doi:10.1016/j.synbio.2026.06.002)
Supplement: Multimedia component 1 [file mmc1.docx]

**Metabolic State-Driven Monitoring and Control of Abnormal Pigment Formation in Sodium Gluconate Fermentation by *Aspergillus niger***

Jingchun Sun^a,b,#^, Yuanyuan Jiang^a,b,#^, Xing Jiang^a,b^, Wei Zhao^d^, Xiwei Tian^a,b,c^, Ju Chu^a,b,c^, Feng Xu^a,b,c,*^

^a^ State Key Laboratory of Bioreactor Engineering, Qingdao Innovation Institute of East China University of Science and Technology, East China University of Science and Technology, Shanghai 200237, China

^b^ National Center of Bio-Engineering & Technology (Shanghai), East China University of Science and Technology, Shanghai 200237, China

^c^ Shanghai Collaborative Innovation Center for Biomanufacturing Technology, East China University of Science and Technology, Shanghai 200237, China

^d^ Shan Dong Fuyang Biological Technology Co., Ltd, Longmen Economic Development Zone, Dezhou Plain, Shandong, 253100, China

* Corresponding author: Feng Xu

# Jingchun Sun and Yuanyuan Jiang have contributed equally to this work.

Tel: +86-21-64253021

E-mail address: fengxu@ecust.edu.cn (Feng Xu)

**Content**

**Figure S1.** Visual comparison of different fermentation batch. (a) Typical fermentation broth color in 200 m^3^ bioreactor. (b) The fermentation broth color of yellow pigment-producing batch.

**Figure S2.** Comprehensive performance benchmarking of various machine learning regression algorithms. (a) Comparison of the Coefficient of Determination (R^2^) for training and testing datasets. (b) Root Mean Squared Error (RMSE) evaluation on the testing set.

**Figure S3.** Interpretation of model input features and assessment of learning performance. (a) Feature importance analysis: Ranking of input variables based on their contribution to the model's predictive output. (b) Learning curve: The evolution of Mean Squared Error (MSE) for training (blue line) and validation (orange line) sets as a function of training sample size.

**Figure S4.** Score plots of the first two latent variables (LVs). (a) Score plot for DCW model. (b) Score plot for Glucose model. (c) Score plot for Product model.

**Figure S5.** Outlier detection and diagnostic analysis for the specific models. (a-c) Hotelling’s T^2^ versus Q-residuals control charts: These plots display the distribution of samples in the multivariate projection space. The horizontal and vertical dashed lines represent the 95% confidence limits (p=0.95). Samples located within the bottom-left quadrant are considered normal, while points outside these boundaries are identified as potential outliers in the input feature space. (a) Model for DCW. (b) Model for glucose concentration. (c) Model for sodium gluconate. (d-f) Williams plots (Standardized Residuals versus Leverage): These plots evaluate the applicability domain and predictive reliability of the regression models. The horizontal dashed lines indicate the residual outliers’ limits, while the vertical dashed line indicates the critical leverage value. Points falling within the rectangular region represent reliable predictions with low influence. (d) Diagnostic plot for the DCW model. (e) Diagnostic plot for the Glucose model. (f) Diagnostic plot for the SG model. Different colors represent different fermentation batches or sample groups used in the dataset.

**Figure S6.** Variable Importance in Projection (VIP) scores for identifying key spectral variables contributing to the PLS models. (a) VIP scores for the Dry Cell Weight (DCW) model. (b) VIP scores for the Glucose (GLC) model. (c) VIP scores for the Sodium Gluconate (SG) model.

**Figure S7.** Kinetic profiles of fermentation parameters under varying concentrations of inorganic salts. (a-c) Effect of potassium dihydrogen phosphate (KH_2_PO_4_) supplementation: The fermentation dynamics were monitored at concentrations of 0.333, 0.5, and 1.0 g/L. (a) Dry Cell Weight (DCW). (b) Pigment accumulation (OD_405_). (c) Residual glucose concentration. (d-f) Effect of magnesium sulfate (MgSO_4_) supplementation: The process was evaluated at 0.19 and 0.333 g/L. (d) Pigment accumulation (OD_405_). (e) Dry Cell Weight (DCW). (f) Residual glucose concentration. Error bars indicate standard deviation (n=3).


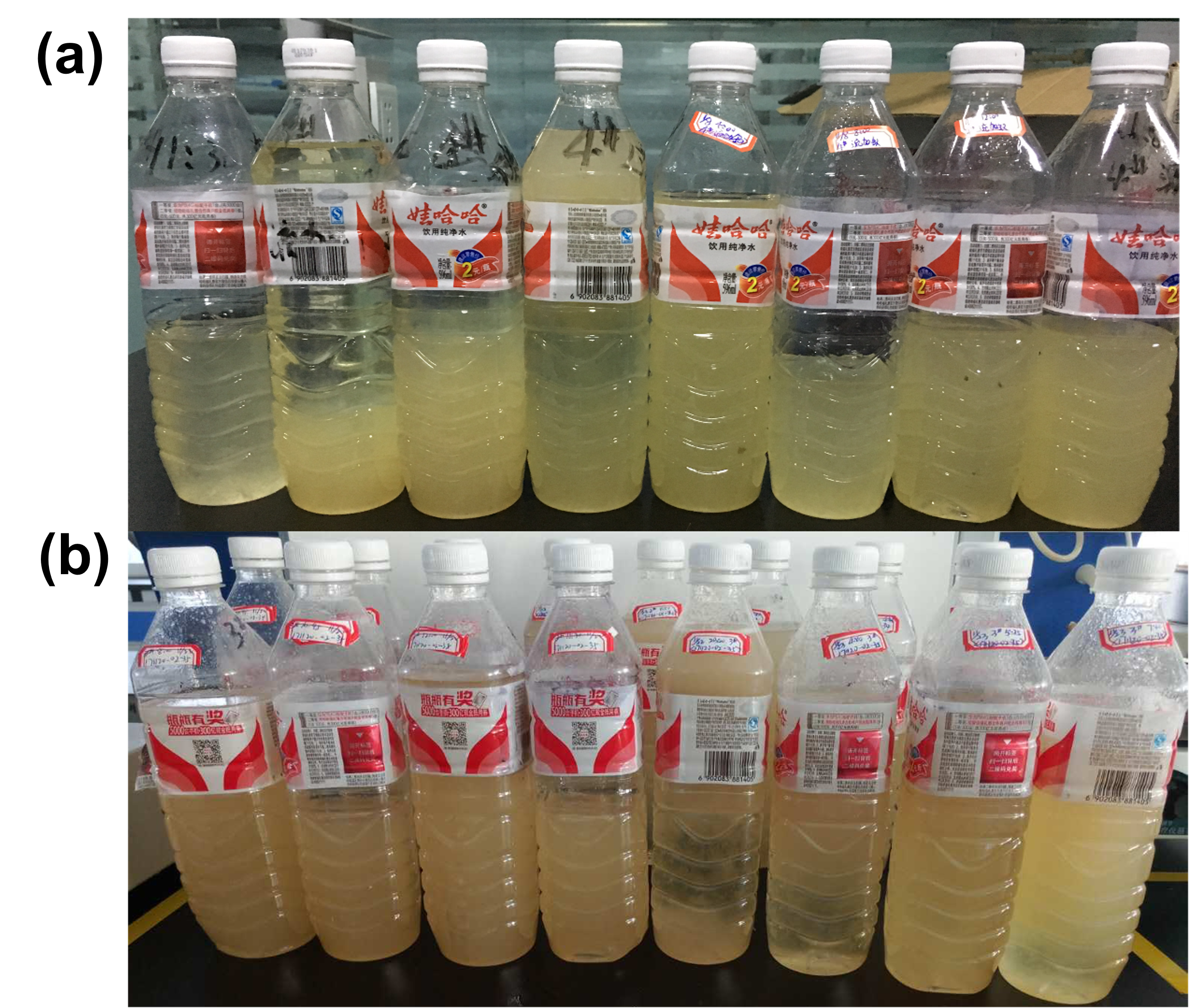


**Figure S1.**


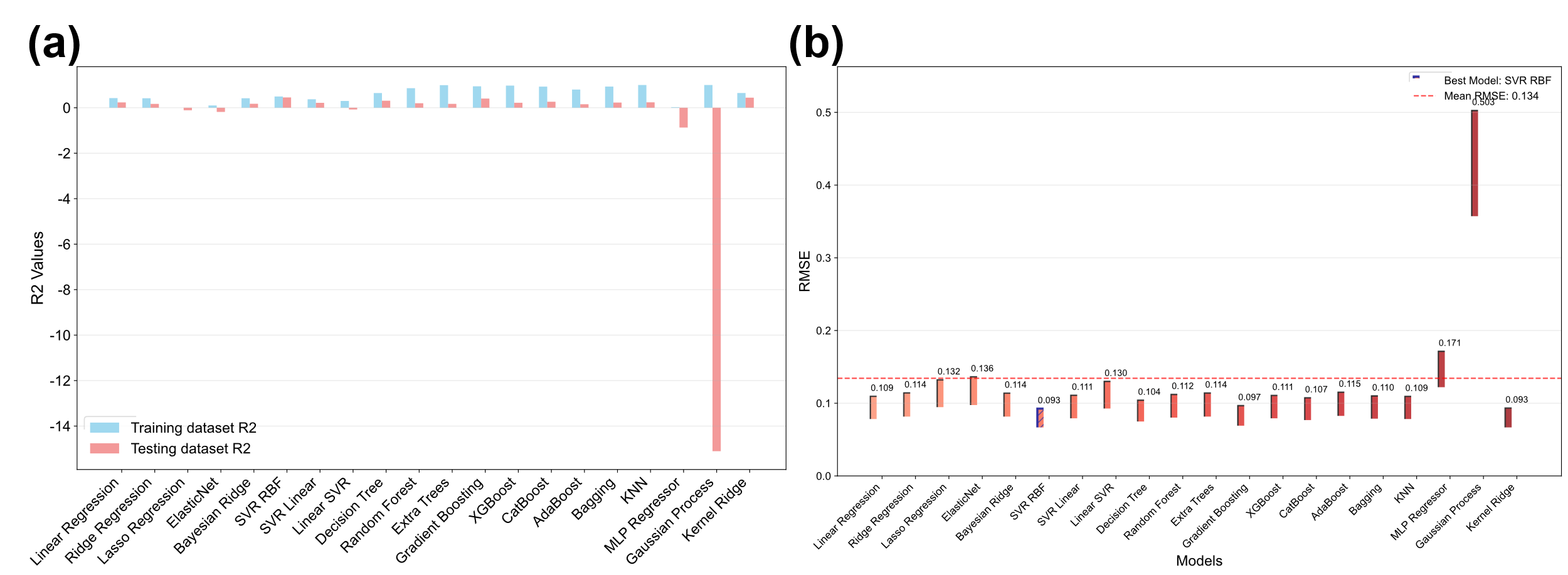


**Figure S2.**


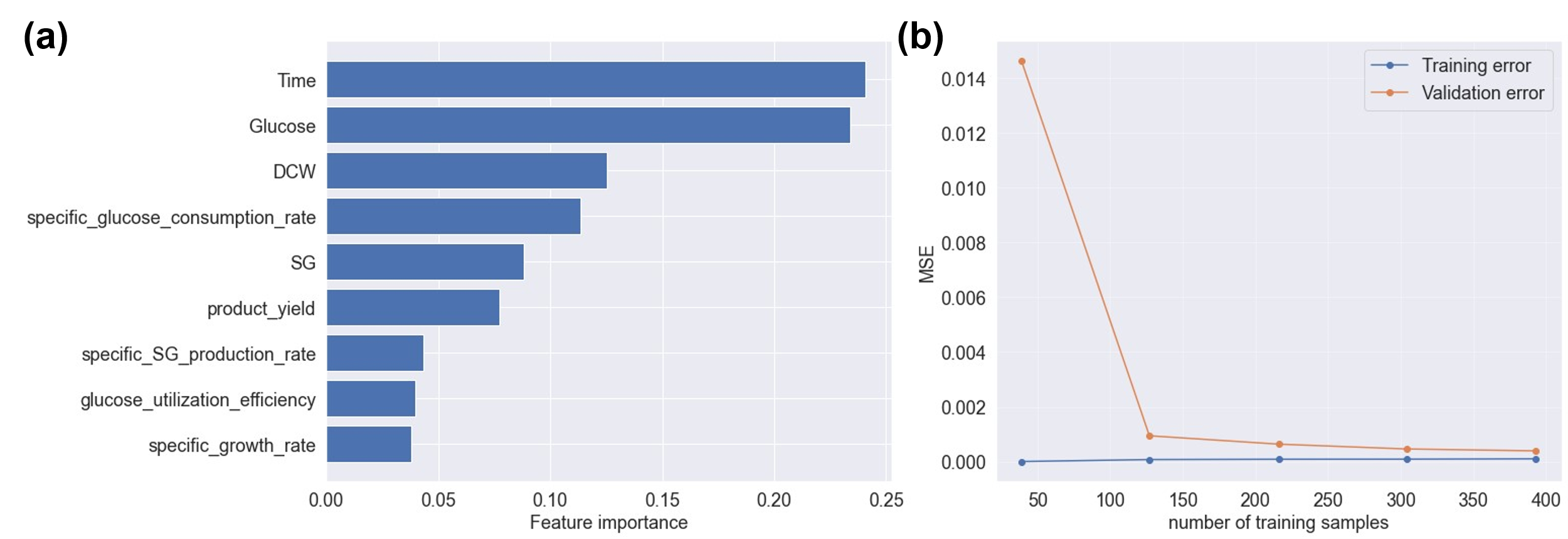


**Figure S3.**


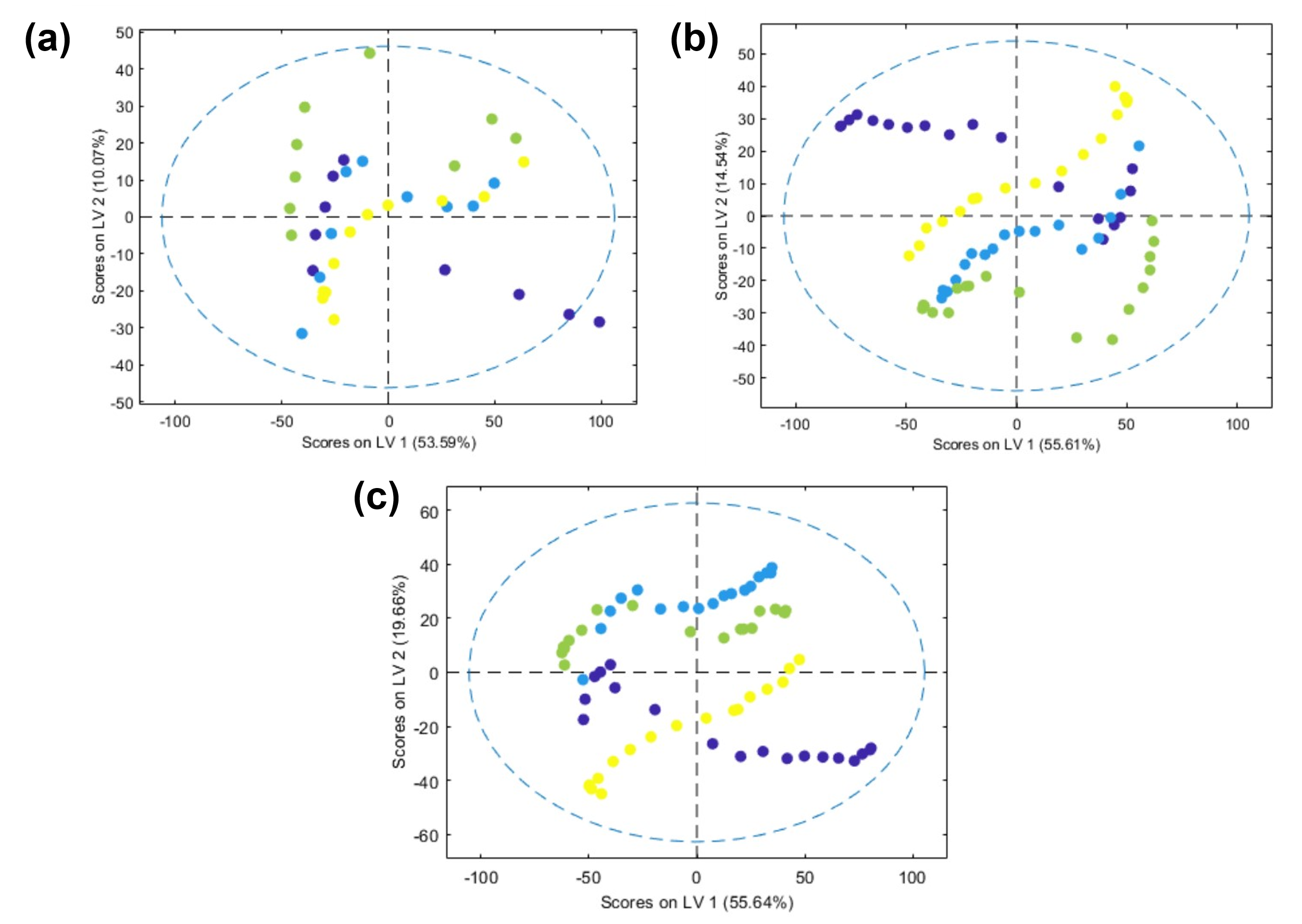


**Figure S4.**


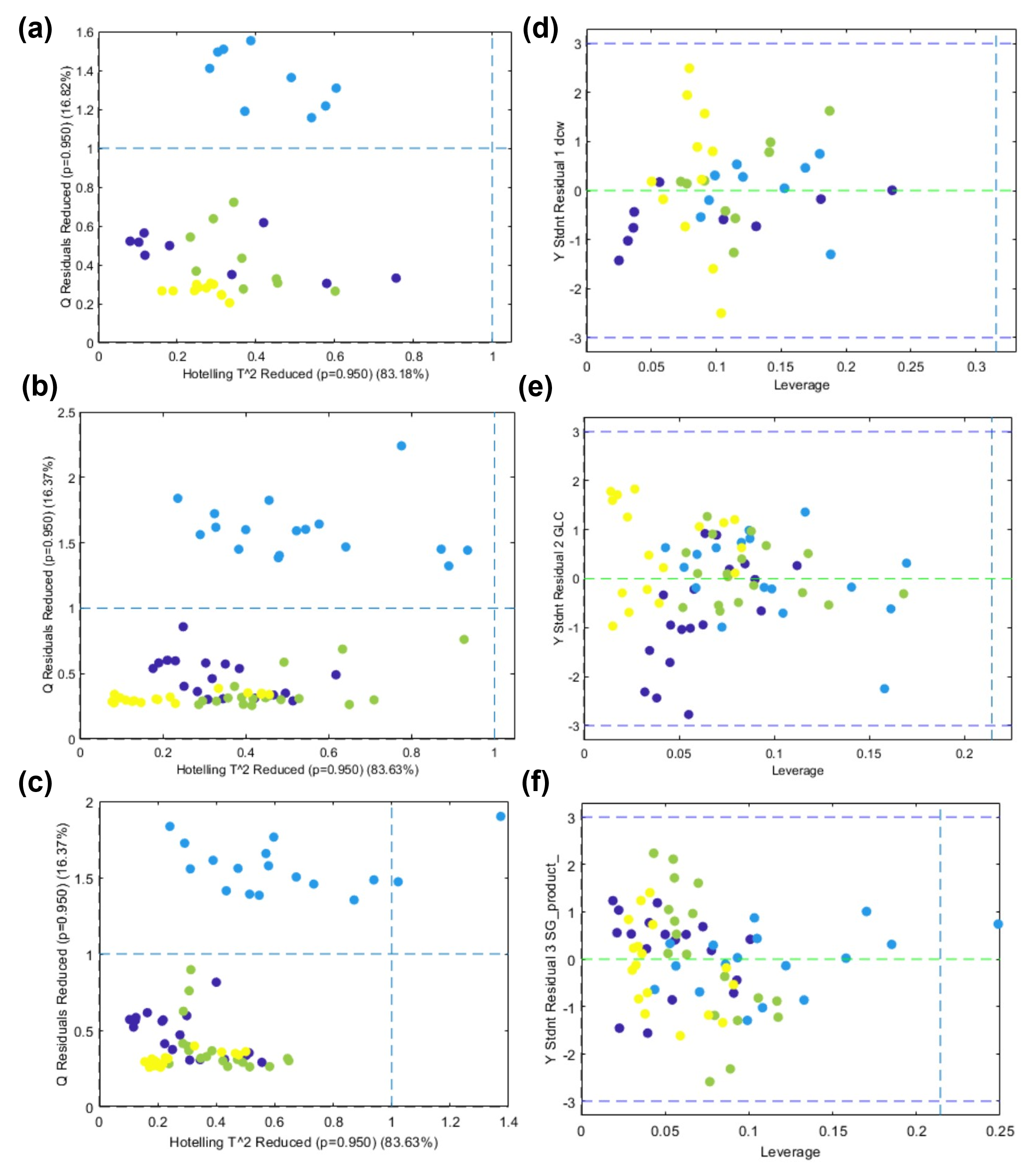


**Figure S5.**


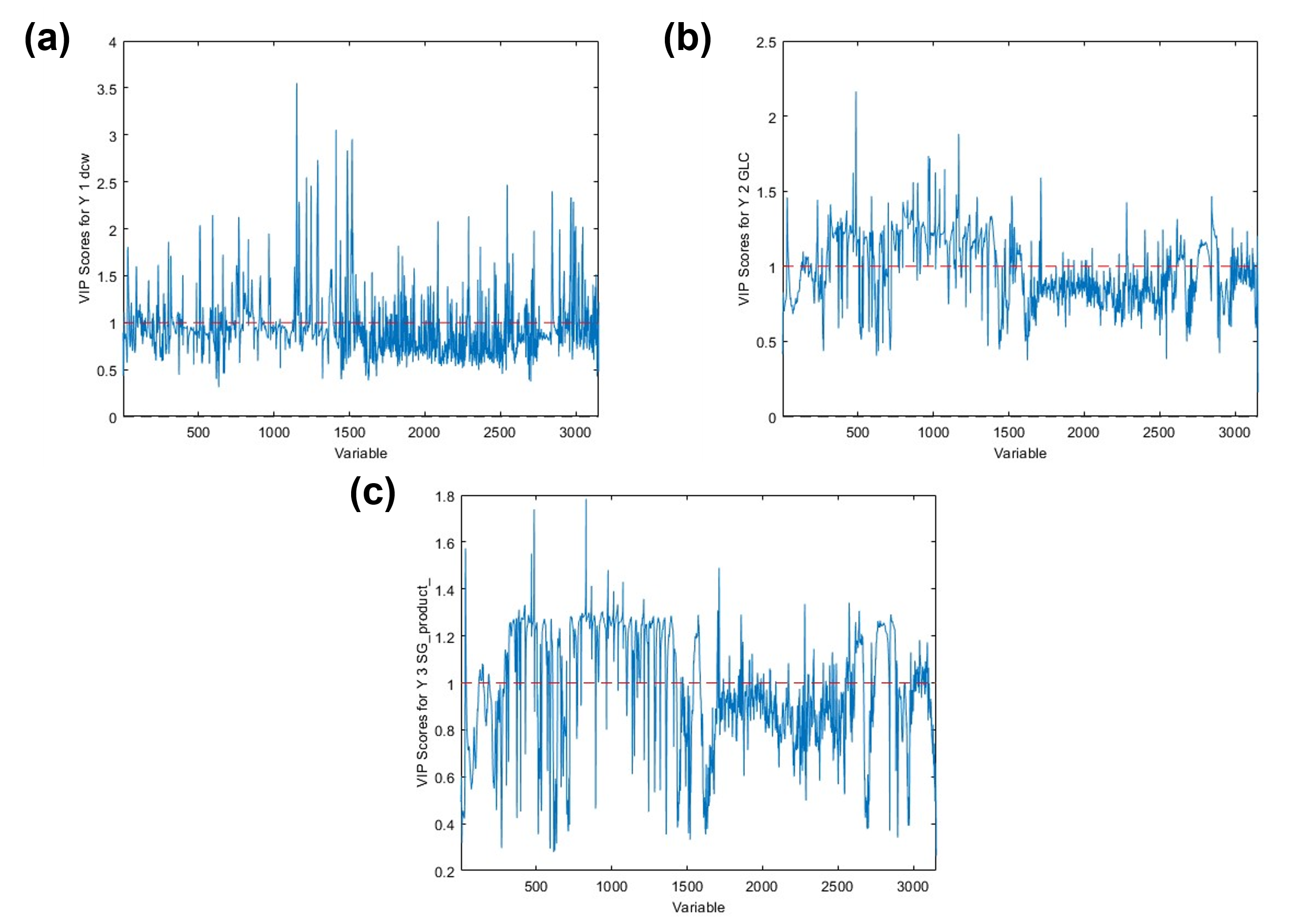


**Figure S6.**


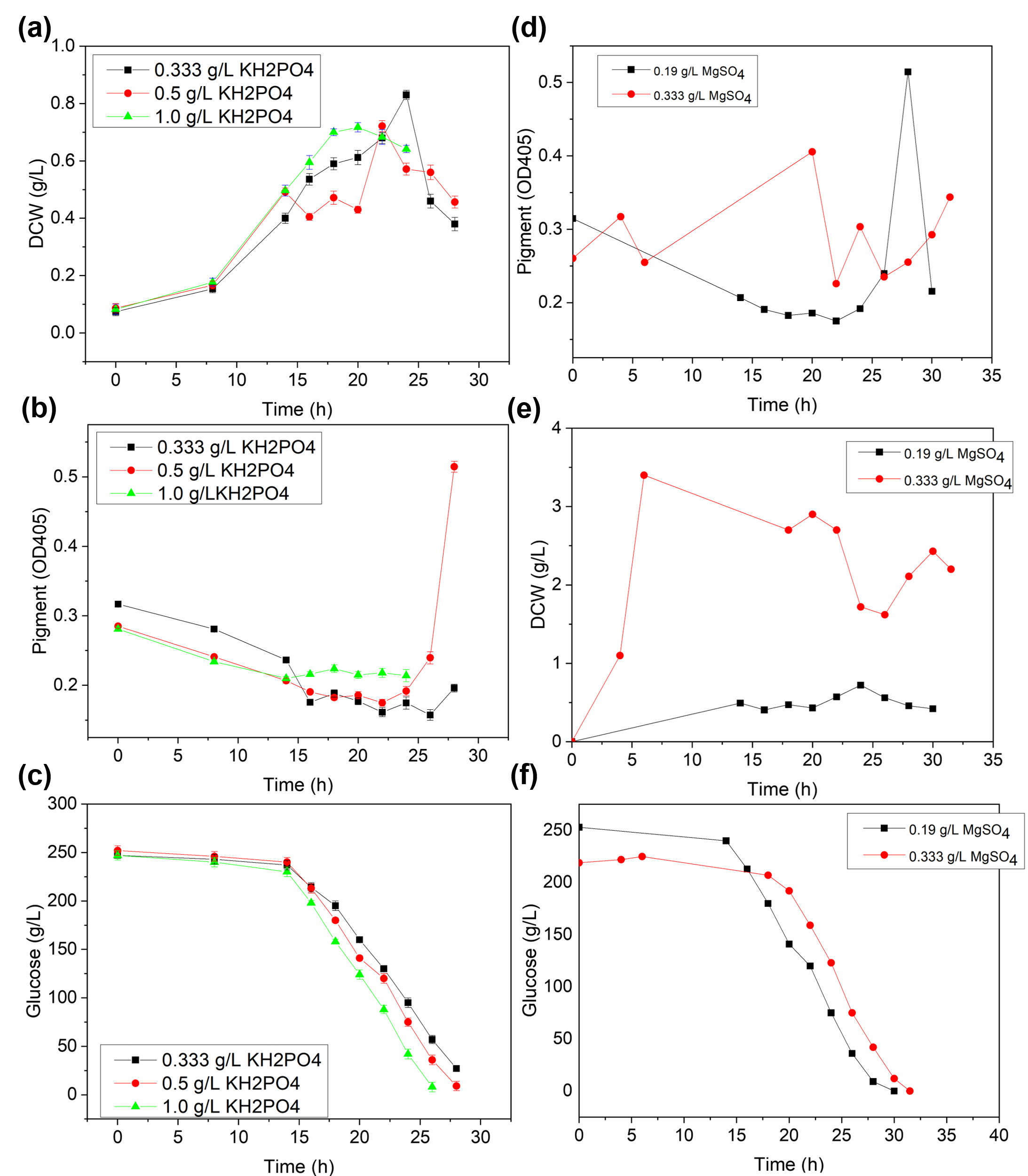


**Figure S7.**
